# Systematic review and meta-analysis: real-world data rates of deep remission with anti-TNFα in inflammatory bowel disease

**DOI:** 10.1186/s12876-021-01883-6

**Published:** 2021-08-03

**Authors:** Omeed Alipour, Alakh Gualti, Ling Shao, Bing Zhang

**Affiliations:** 1 Department of Medicine, Division of Gastroenterology, University of Washington, 1959 NE Pacific Street, Box 356424, Seattle, WA 98195 USA; 2 Department of Medicine, Division of Gastroenterology and Hepatology, University of California San Francisco – Fresno, 155 N Fresno Street, Fresno, CA 93721 USA; 3 Department of Medicine, Division of Gastrointestinal and Liver Diseases, Keck School of Medicine, University of Southern California, 2011 Zonal Avenue, HMR 101, Los Angeles, CA 90033 USA; 4 Department of Medicine, Division of Gastroenterology and Hepatology, University of California San Francisco, 513 Parnassus Avenue, Room S-237, San Francisco, CA 94143 USA

**Keywords:** Remission, Inflammatory bowel disease, Crohn’s disease, Ulcerative colitis, Tumor necrosis factor-a inhibitor

## Abstract

**Background:**

Deep remission (DR) is a treatment target in IBD associated with reduced hospitalization and improved outcome. Randomized control trial (RCT) data demonstrates efficacy of anti-TNFα agents in achieving DR; however, real-world data (RWD) can provide information complementary to RCTs, specifically regarding treatment duration. In this systematic review with meta-analysis, we use real-world data (RWD) to determine rates of DR in IBD treated with anti-TNFα.

**Methods:**

We completed a systematic search of MEDLINE and EMBASE on July 8, 2019 with review of major gastrointestinal conference abstracts from 2012 to 2019. Studies utilizing RWD (data not from phase I-III RCTs) of adult IBD patients treated with anti-TNFα agents were included. DR was defined by clinical and endoscopic remission at minimum. DR was assessed at 8 weeks, 6 months, 1 year, and 2 years. Risk of bias was assessed with the Newcastle Ottawa Scale.

**Results:**

29,033 publications were identified. Fifteen publications, nine manuscripts and six conference abstracts, were included encompassing 1212 patients (769 Crohn’s disease-CD, 443 ulcerative colitis-UC), and analyzed using Comprehensive Meta-Analysis. Rate of DR was 36.4% (95% CI 12.6–69.4%) at 8 weeks, 39.1% (95% CI 10.4–78%) at 6 months, 44.4% (95% CI 34.6–54.6%) at 1 year, and 36% (95% CI 18.7–58%) at 2 years. DR in CD at 1 year was 48.6% (95% CI 32.8–64.7%) and in UC was 43.6% (95% CI 32.8–55.1%).

**Conclusions:**

The rate of DR was highest after 1 year of therapy, in nearly 45% of IBD patients treated with anti-TNFα. Similar rates were achieved between patients with UC and CD. The findings highlight the efficacy of anti-TNFα in real-world setting. Future studies using RWD can determine efficacy of newer IBD therapeutics in routine clinical practice.

**Supplementary Information:**

The online version contains supplementary material available at 10.1186/s12876-021-01883-6.

## Background

Deep remission (DR) is a proposed treatment target in inflammatory bowel disease (IBD) that is increasingly being used as a benchmark in efficacy studies and randomized controlled trials (RCT) [1]. The most common definition for DR is concurrent clinical remission (CR) and endoscopic remission (ER) or mucosal healing (MH) [2]. DR is associated with longer periods of durable remission, improvement in quality of life, reduced hospitalization, and a decreased rate of surgical complications [3–6]. Therefore, there is great interest in determining the rate of achieving DR with various treatment strategies.

Recent meta-analyses have examined the rate of achieving DR with anti-TNFα agents in randomized controlled trials (RCTs) among ulcerative colitis (UC) patients [7], but none have evaluated DR in a real-world environment or in patients with Crohn’s disease (CD). Differences between the efficacy of a drug’s performance during a clinical trial and its effectiveness during use in everyday clinical practice has been described as the “efficacy-effectiveness gap” [8]. RCTs, though the ideal study design to demonstrate effectiveness and safety of a medication, are carried out in selective and controlled manner leading to high internal validity, but leaving uncertainty about their generalizability for an ethnically diverse and heterogenous population [9]. This possible lack of generalizability has also been demonstrated within the IBD population [10], and therefore creates a role for real world data (RWD) to fill [11].

In this systematic review with meta-analysis, we aim to provide complementary information by using RWD to determine rates of deep remission in IBD with anti-TNFα agents in clinical practice. Additionally, we perform sub-analyses to provide the rates of DR with anti-TNFα separately in patients with CD and UC. Furthermore, we explored the treatment duration at which DR is most likely to be seen, and the rate of DR in patients not previously treated with anti-TNFα.

## Methods

The current study, including abstract and manuscript content, was completed in accordance with the PRISMA statement and checklist (Additional file 1: Tables S1, S2) [12].

### Data sources and searches

We completed a systematic search of MEDLINE and EMBASE up to July 8, 2019 (see Additional file 2: Text/Appendix 1 for search strategy), using the following search terms: (“inflammatory bowel disease” OR “IBD” OR “crohn*” OR “ulcerative colitis” OR “UC” or “colitis”) AND (“mucosal healing” OR “deep remission” OR “complete remission” OR “full remission” OR “endoscopic remission”). This search was conducted without restrictions on year or language. We manually searched through abstracts presented at major national and international gastrointestinal conferences from 2012 to 2019 (Digestive Disease Week, United European Gastroenterology Week, European Crohn’s and Colitis Organization, the American College of Gastroenterology Annual Scientific Meeting, Advances in Inflammatory Bowel Diseases, and the Crohn’s and Colitis Congress). The reference sections of manuscripts included were also reviewed for additional studies to be evaluated for inclusion. Two authors (OA and AG) independently conducted this review. A third author (BZ) reviewed studies not agreed upon for inclusion. A cursory updated search of MEDLINE and EMBASE was performed by one author (BZ) from July 8, 2019 to April 25, 2021 (see Additional file 2: Text/Appendix 1). This systematic review was not pre-registered and a prior review protocol was not prepared.

### Selection criteria

We included studies that presented real world data (RWD)/real world evidence (RWE), defined as all health data except those collected in a conventional phase I, II, or III RCT setting, including non-randomized controlled group studies. We included studies examining adults (18 years or older) with inflammatory bowel disease treated with anti-TNFα agents until the achievement of “deep remission” (DR), defined as at least a combination of clinical remission and mucosal healing/endoscopic remission [2]. Search results were carefully reviewed to identify remission targets consistent with common definitions of deep remission given many did publications did not explicitly state the term “deep remission” as an end point.

Case reports, case series, randomized trials, and non-English studies were excluded. Studies that did not define DR or did not identify components of DR to include at least clinical and endoscopic remission were excluded. Studies with a pediatric population were excluded to maintain a focus on adult patients.

The primary outcome was real-world rates of DR with anti-TNFα agents for the treatment of IBD at intervals of 8 weeks, 6 months, 1 year and 2 years after starting anti-TNFα. Secondary outcomes included rates of DR among UC and CD at 1 year after starting anti-TNFα, the rates of DR in patients naïve to, or not previously treated with, anti-TNFα, and the rates of DR with infliximab.

### Data extraction and risk of bias assessment

Two authors (OA and AG) independently extracted the following data onto a data collection form: first author’s name, last author’s name, publication year, country, single or multiple institutions, study design, type of IBD, type of anti-TNFα used, concomitant or maintenance therapy, definition of deep remission, definition of mucosal healing/endoscopic remission, definition of clinical remission, and the number of participants who achieved deep remission at pre-determined time points (8 weeks, 6 months, 1 year, and 2 years).

All studies were deemed cohort studies based on the intervention of interest (treatment with anti-TNFα agents). Risk of bias was assessed independently by two authors (OA and AG) using the Newcastle-Ottawa Scale [13]. Any inconsistencies between the authors’ scores were discussed and resolved. Out of nine possible stars, studies were considered at high risk of bias if they received 0–3 stars, intermediate risk if 4–6 stars, and low risk if 7–9 stars. The quality of evidence was determined based on the GRADE (Grading of Recommendations Assessment, Development, and Evaluation) system [14]. Quality of evidence ranges from “high” to “moderate” to “low” and “very low” based on the effect future research is expected to have and the certainty of the findings.

### Data synthesis

To account for anticipated inherent heterogeneity in the designs of the included studies (for example, retrospective versus prospective, definitions of deep remission, anti-TNFα agents used, patient populations, etc.), pooled event rates and corresponding 95% confidence intervals (95% CI) were calculated using the random-effects model per DerSimonian and Laird and inverse variance method for dichotomous outcomes [15]. Between-study heterogeneity was assessed with the chi-square test with significance defined as p < 0.1, and the I^2^ test at > 50% [16]. Publication bias was assessed with funnel plot and Egger test (Additional file 4: Figure S2). All analyses were performed using Comprehensive Meta-Analysis (version 3; Biostat, Englewood, NJ, USA, 2013).

## Results

### Search results

**Fig. 1:**
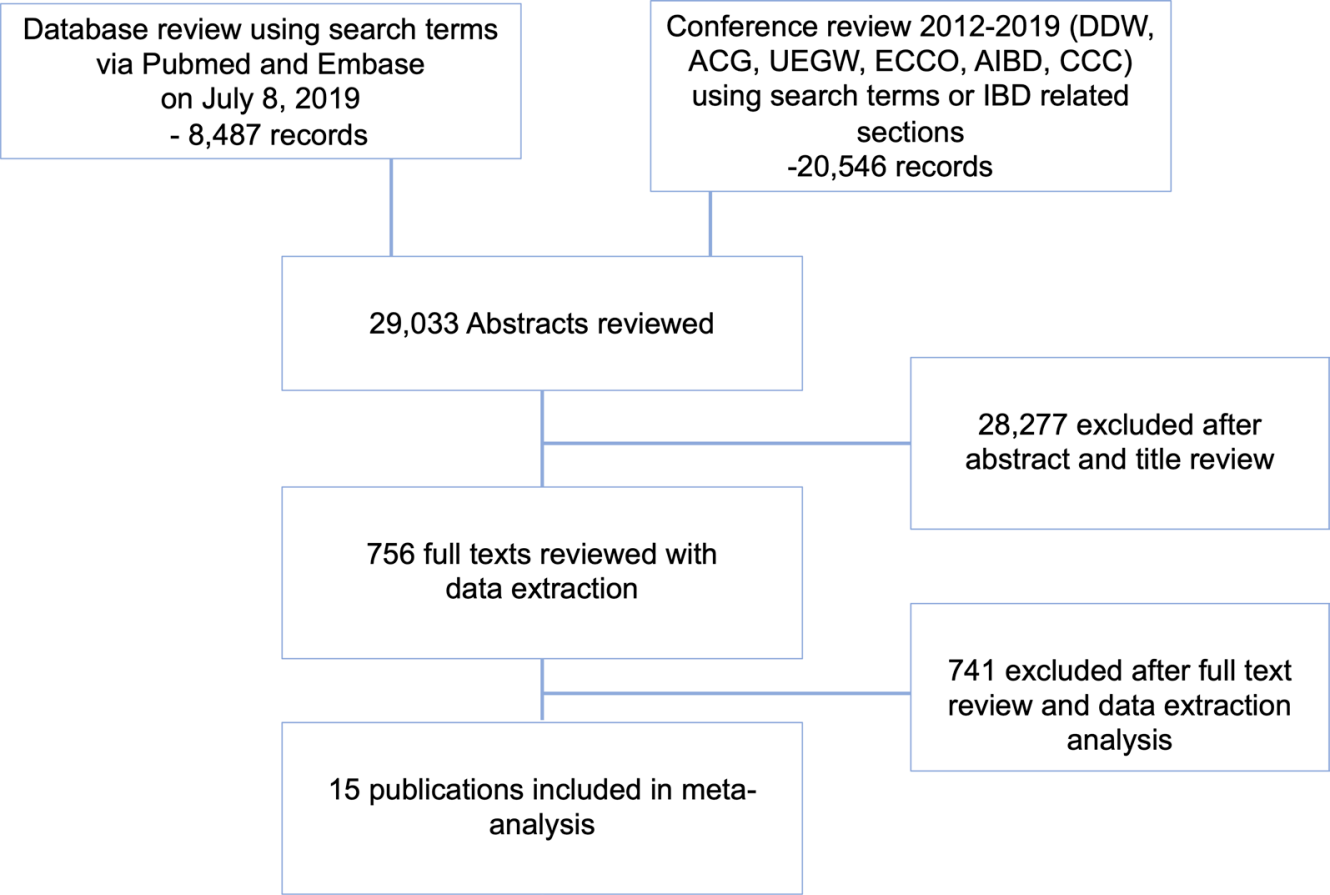
PRISMA diagram

The search strategy identified 29,033 publications. After a review of titles, abstracts, and exclusion of duplicates, 756 articles underwent thorough review (Fig. 1). Application of the exclusion criteria yielded fifteen studies (9 manuscripts, 6 conference abstracts), encompassing a total of 1212 patients (Table 1) [17–31]. A diagnosis of CD was captured for 769 patients, and a diagnosis of UC was provided for 443 patients. A cursory updated search of MEDLINE and EMBASE using the same strategy as above from July 8, 2019 to April 25, 2021 yielded 1722 new publications (596 MEDLINE, 1126 EMBASE). 93 publications underwent thorough review; none included data meeting inclusion criteria. Most excluded studies were not eligible for inclusion because they did not meet the minimum criteria for deep remission, length of follow up, or sample size.

**Table 1.** Characteristics of studies included in meta-analysis

Study	Country	Study design	DR criteria	Patients evaluated for DR		Anti-TNF	Concomitant therapeutics	Time of DR assessment	Prior anti-TNF use
				UC	CD				
Sebkova 2012	Czech Republic	Retro	CDAI <150, no mucosal ulcerations	–	60	IFX^†^ or ADA^‡^	IMM^§^ (not specified, used in 30/60)	1 year	Yes (17/60)
Vadan 2012	Romania	Pro	CDAI <150, no mucosal ulcerations	–	49*	IFX^†^	IMM^§^ (5/49)	1 year	No
Vadan 2013	Romania	Pro	CDAI <150, no mucosal ulcerations		49*	IFX^†^	IMM^§^ (5/49)	6 months	No
De Vos 2013	Belgium	Pro	Partial Mayo <3, endo Mayo 0	87	–	IFX^†^	AZA^¶^ (60/87)	1 year	No
Molander 2013	Finland	Retro	No clinical symptoms, SES-CD 0–2, endo Mayo 0–1	69	183	IFX^†^ or ADA^‡^	AZA^¶^ (140/252), 6MP (23/252), MTX (22/252)	2 year	Not specified
Dai 2014	China	Pro	CDAI <150, Mayo <2, SES-CD 0–3, endo Mayo 0	107	109	IFX^†^	IMM^§^ (66/109 CD, 34/107 UC)	1 year	Yes (17 CD, 3 UC)
Echarri 2015	Spain	Pro	HBI <5, SES-CD 0–2	–	64	ADA^‡^	AZA^¶^ (44/68), steroids (56/68)	6 months, 1 year, 2 year	No
Yu 2015	China	Retro	CDAI <150, SES-CD 0–2	–	106	IFX^†^	AZA^¶^ (49/106)	8 weeks	No
Magro 2016	Portugal	Pro	Mayo 0–2, endo Mayo 0–1, Geboe’s score <4	20	–	IFX^†^	AZA^¶^ (17/20)	8 weeks, 6 month, 1 year	No
Pineton de Chambrun 2016	France	Retro	Clinical physician assessment, no ulcerations	–	67	IFX^†^	AZA^¶^ (26/67)	2 year	No
Prymak 2016	Ukraine	Pro	CAI and UCEIS (not defined)	51	–	IFX^†^	Mesalamine + steroids (25/51)	8 weeks	No
Zhang 2016	China	Pro	CDAI <150, no ulcerations	–	22	IFX^†^	None	6 month, 1 year, 2 year	No
Kaymak 2018	Switzerland	Retro	HBI <5 or CDAI <150, Fcal <150 × 2 years, no ulcerations (endoscopic and histologically)	–	109	IFX^†^	Not specified; steroid refractory	2 year	Yes (11/109 prior ADA)
Kumar 2018	UK	Retro	CR + ER (undefined)	56	–	Golimumab	IMM^§^ (36/56)	1 year	Not specified
Munoz-Villafranca 2018	Spain	Pro	pMayo 0–2, endo Mayo 0–1	53	–	ADA^‡^	IMM^§^ (38/53)	8 weeks, 1 year	No

^†^Infliximab, ^‡^Adalimumab, ^§^Immunomodulators, ^¶^Azathioprine

All studies originated in Europe with the exception of Yu 2015, Dai 2014, and Zhang 2016 [19, 21, 22]. Nine studies were prospective in design [17, 19, 20, 22, 24, 25, 28–30], and seven were carried out at multiple institutions [17, 18, 20, 21, 24, 25, 31]. No phase IV trials were identified for inclusion. Two studies defined deep remission (DR) beyond the minimum criteria of clinical remission and endoscopic remission—Magro 2016 included histologic remission defined as a Geboe’s score < 4, and Kaymak 2018 supplemented both histologic remission and 2 years of biochemical remission (fecal calprotectin < 150) [23, 25]. Two conference abstracts used the same cohort and reported rates of DR at different time points, therefore this cohort was only counted one time [28, 29]. Ten studies featured only infliximab (IFX) [17, 19, 21–23, 25, 27–30], two used only adalimumab (ADA) [20, 24], one study assessed golimumab [31], and two studies incorporated both IFX and ADA [18, 26]. Pineton de Chambrun 2016 reported that 65% of DR patients received concomitant therapy with AZA, whereas only 28% of their non-DR group was receiving concomitant AZA. Other studies did not specify concomitant therapy use. Most included studies did not specify the number of cases with perianal or fistulizing disease, precluding additional statistics for this sub-population of patients. Similarly, the majority of studies evaluating CD did not clearly indicate if patients were pre-operative, though most were TNF naïve. No studies reported use of biosimilar agents. The heterogeneities of studies are reported (Additional file 1: Table S3) with I^2^ values for all analyses over 72%, consistent with considerable heterogeneity.

### Quality of studies and risk of bias

The Newcastle Ottawa Scale (NOS) was used to evaluate and assign a point value to each study for quality and risk of bias (Additional file 1: Table S4) [13]. Studies received a point for “adequacy of follow up of cohorts” if their reported outcomes accounted for attrition. All included studies received between five or six points on the NOS, suggesting that they carried an intermediate risk of bias. Two studies, De Vos 2013 and Zhang 2016, included patients already in clinical remission, additional sensitivity analyses were run with these studies excluded (Additional file 3: Figure S1) [17, 22].

### Achieving deep remission at 8 weeks, 6 months, 1 year, and 2 years

**Fig. 2:**
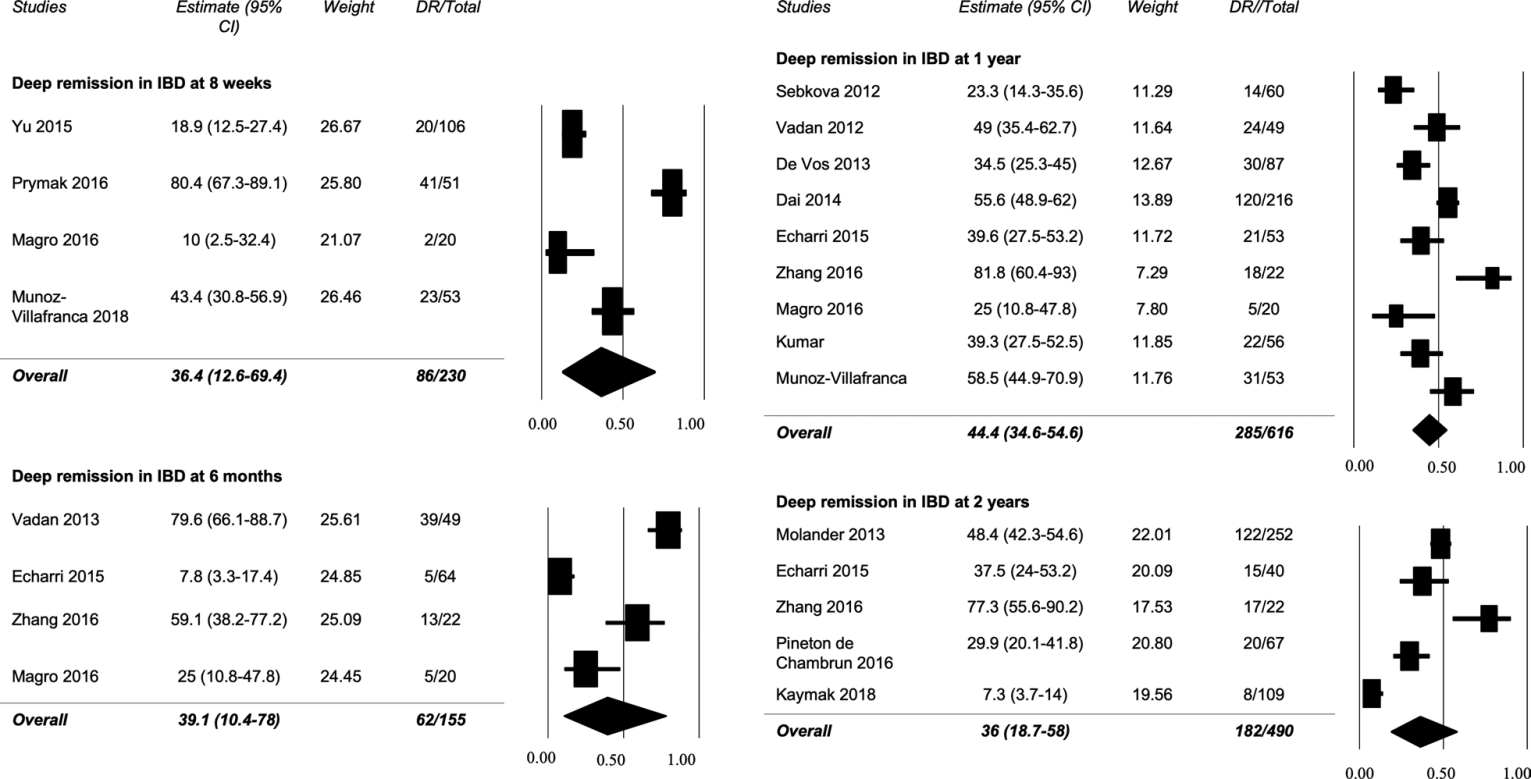
Rates of deep remission in IBD at 8 weeks, 6 months, 1 year, and 2 years

Four studies reported a combined 36.4% (95% CI 12.6–69.4%) rate (86/230 patients) of achieving DR at 8 weeks [21, 24, 25, 30]. Four studies reported the rate of DR at 6 months [20, 22, 25, 29], with 39.1% (95% CI 10.4–78.0%), or 62/155 patients, achieving the treatment target (Fig. 2). Of these four studies, Zhang 2016 only included patients already in clinical remission [22]. Sensitivity analysis with Zhang 2016 removed demonstrated a 32.8% rate of deep remission at 6 months (Additional file 3: Figure S1). Funnel plots (Additional file 4: Figure S2) and Egger’s test for both 8 weeks and 6 months did not detect publication bias (8 week: Egger’s t-value 0.056, p = 0.480; 6 month: Egger’s t-value = 2.002, p = 0.091). Heterogeneity with these analyses reflected as an I^2^ value were 94% and 93.7%, respectively, suggesting considerable heterogeneity of included studies (Additional file 1: Table S3).

Nine studies reported the rate of DR at 1-year follow-up [17, 19, 20, 22, 24–26, 28, 31], with 44.4% (95% CI 34.6–54.6%) of patients (285/616) achieving DR. Five studies reported rates of DR at 2 years, with 36% (95% CI 18.7–58%) of patients (182/490) in DR (Fig. 2) [18, 20, 22, 23, 27]. The only two studies with five points in the NOS were in the DR at 2 years analysis, introducing higher risk of bias and uncertainty in this analysis compared to the 8 week, 6 month, and 1 year analyses. For 1 year, De Vos 2013 and Zhang 2016 only included patients already in clinical remission [17, 22]. Sensitivity analysis with Zhang 2016 and De Vos 2013 removed demonstrated a 42.3% rate of deep remission at 1 year, and sensitivity analysis with Zhang 2016 removed at 2 year analysis had a deep remission rate of 27.8% (Additional file 3: Figure S1). Funnel plots (Additional file 4: Figure S2) and Egger’s test at one-year and two-years did not demonstrate publication bias (1 year: Egger’s t-value = 0.703, p = 0.252; 2 years: Egger’s t-value = 0.673, p = 0.275). Heterogeneity within these analyses, reflected as an I^2^ value, were 80.6% and 92.6%, respectively, suggesting considerable heterogeneity of included studies (Additional file 1: Table S3). The GRADE quality of evidence for this analysis is ‘low’.

### Achieving deep remission in Crohn’s disease and ulcerative colitis

**Fig. 3:**
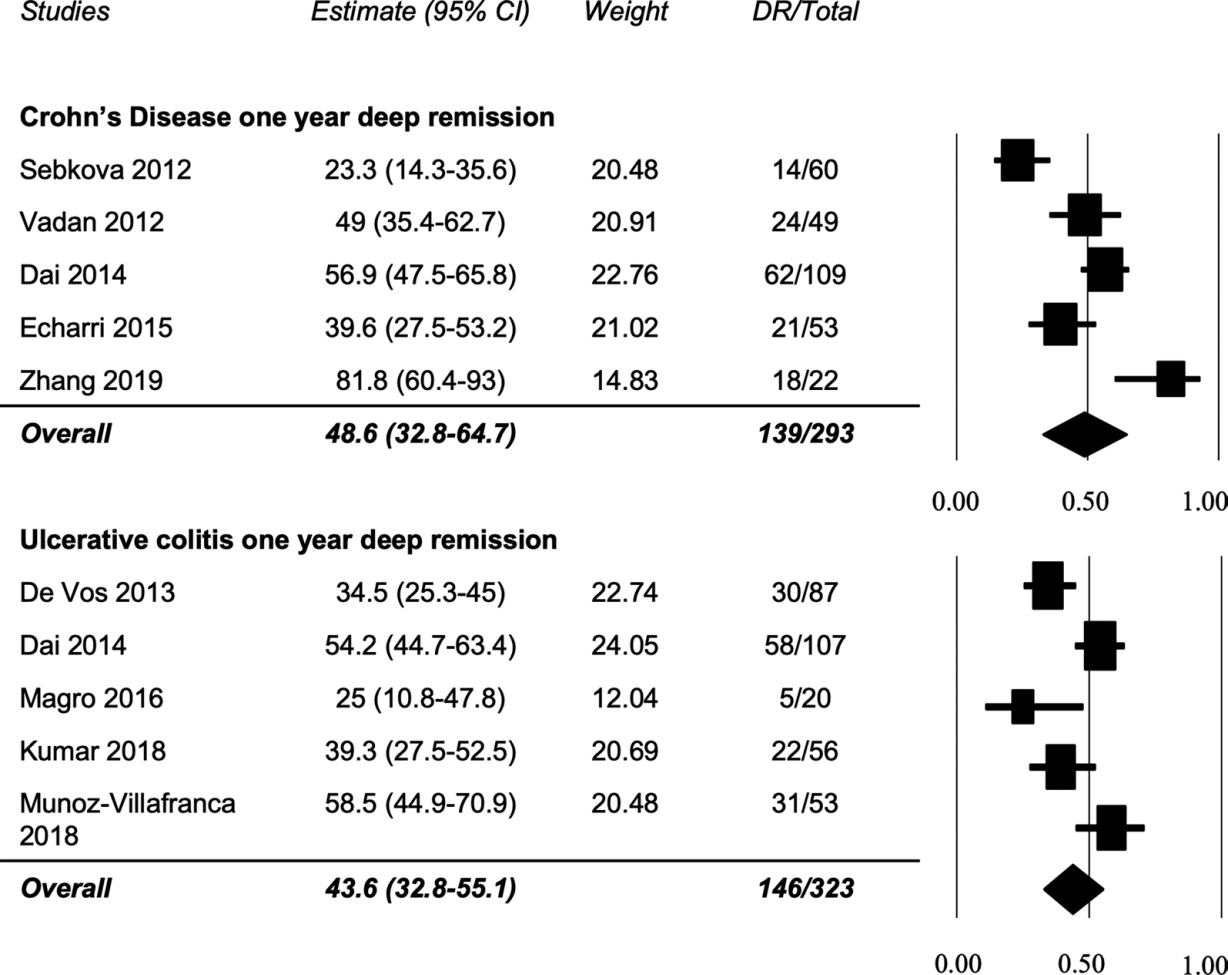
Rates of deep remission in Crohn’s disease and ulcerative colitis at 1 year

*Crohn’s Disease (CD)*: Ten studies reported rates of DR in 769 patients with CD between 8 weeks and 2 years [18–23, 26–29]. At 8 weeks, one study reported 18.9% DR [21]. DR at 6 months was reported by three studies to be 7.8% (Echarri 2015), 59.1% (Zhang 2016), and 79.6% (Vadan 2013) [20, 22, 29]. DR at 2 years was reported by five studies to be 43.2% (Molander 2013), 37.5% (Echarri 2015), 77.3% (Zhang 2016), 7.3% (Kaymak 2018), and 29.9% (Pineton de Chambrun 2016) [18, 20, 22, 23, 27]. DR in CD was reported at 1 year by five studies (Fig. 3) and found to be 48.6% (95% CI 32.8–64.7%) in 139/293 patients [19, 20, 22, 26, 28]. Sensitivity analysis with Zhang 2016 removed resulted in a 42.1% rate of deep remission (Additional file 3: Figure S1). Funnel plot and Egger’s test did not demonstrate publication bias. The I^2^ value for this analysis was 84.8% consistent with considerable heterogeneity (Additional file 1: Table S3). The GRADE quality of evidence for this analysis is ‘low’.

*Ulcerative Colitis (UC)*: Seven studies reported the rate of DR in 353 UC patients between 8 weeks and 2 years [17–19, 24, 25, 30, 31]. DR at 8 weeks was reported in three studies to be 43.4% (Munoz-Villafranca 2018) [24], 10% (Magro 2016) [25], and 80.4% (Prymak 2016) [30]. DR at 6 months was reported in one study (Magro 2016) to be 25% [25]. DR at 2 years was reported in one study (Molander 2013) to be 62.3% [18]. DR in UC was reported at one-year by five studies (Fig. 3) and found to be 43.6% (95% CI 32.8–55.1%) in 146/323 patients [17, 19, 24, 25, 31]. Sensitivity analysis with De Vos 2013 removed resulted in a 46.6% deep remission rate at 1 year (Additional file 3: Figure S1). Funnel plot and Egger’s test did not demonstrate publication bias. The I^2^ value for this analysis was 73.3% which may represent substantial heterogeneity (Additional file 1: Table S3). The GRADE quality of evidence for this analysis is ‘low’.

### Deep remission in biologic naïve patients

**Fig. 4:**
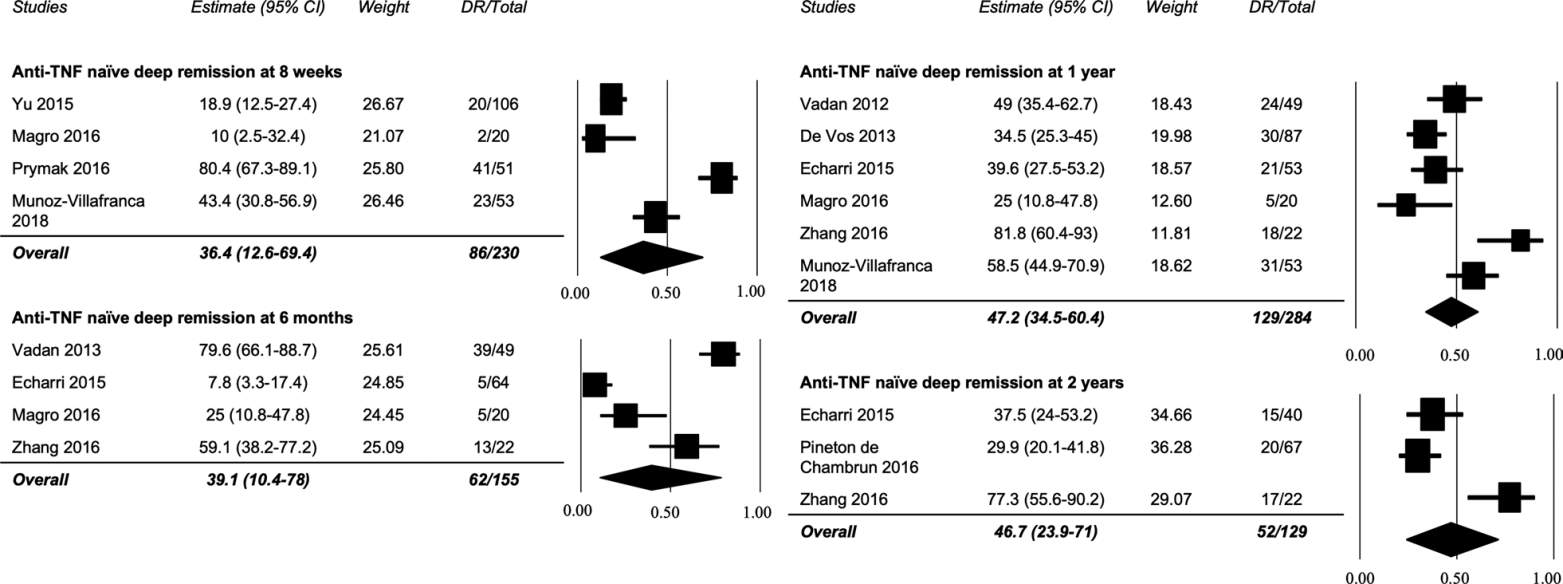
Rates of deep remission in IBD in anti-TNF naïve patients at 8 weeks, 6 months, 1 year, and 2 years

Ten studies specifically indicated that patients were naïve to, or not previously treated with, biologic treatments [17, 20–22, 24, 25, 27–30]. Rates of DR in biologic naïve patients (Fig. 4) was 36.4% (95% CI 12.6–69.4%) in 86/229 patients at 8 weeks in four studies [21, 24, 25, 30], 39.1% (95% CI 10.4–78%) in 62/155 patients at 6 months in four studies [20, 22, 25, 29], 47.2% (95% CI 34.5–60.4%) in 129/284 patients at 1 year in six studies [17, 20, 22, 24, 25, 28], and 46.7% (95% CI 23.9–71%) in 52/129 patients at 2 years in three studies [20, 22, 27]. Funnel plot and Egger’s tests did not demonstrate publication bias except for 2 years (Egger’s t-value = 8.607, p = 0.037). The I^2^ value for these analyses was 94%, 93.7%, 76.3%, and 84.8% (p < 0.05), respectively, consistent with higher heterogeneity (Additional file 1: Table S3). The GRADE quality of evidence was determined to be ‘low’.

### Deep remission in patients treated with infliximab

**Fig. 5:**
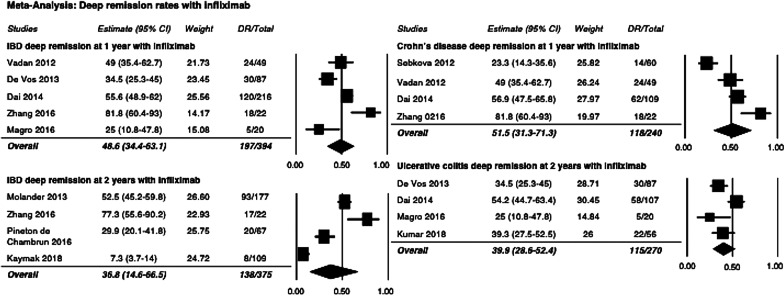
Rates of deep remission in IBD and CD/UC sub-categories with infliximab

The majority of studies primarily included patients treated with infliximab (IFX), therefore additional analyses excluding studies which did not utilize infliximab were conducted to determine rates of DR with IFX. Meta-analysis excluding Sebkova 2012, Kumar 2018, Echarri 2015, and Munoz-Villafranca 2018 demonstrated an IBD deep remission rate of 48.6% at 1 year (Fig. 5). Sensitivity analysis at 2 years with the non-IFX studies Echarri 2015 removed, and excluding non-IFX cases from Molander 2013, resulted in a deep remission rate of 36.8% (Fig. 5). Sensitivity analysis with Echarri 2015 removed found a 51.5% rate of deep remission in CD at 1 year in patient’s receiving infliximab (Fig. 5). Analysis of deep remission in UC at 1 year demonstrated a rate of 39.9% with non-infliximab studies removed (Fig. 5). The I^2^ value for DR with IFX at one and 2 years was 82.4% and 94.8%, respectively. The heterogeneity value for DR with IFX only in CD and UC at 1 year was 82.8% and 72.1%, respectively. The GRADE quality of evidence was determined to be ‘low’ for this analysis.

## Discussion

The ongoing development of novel targeted therapeutics has improved our ability to achieve clinical and endoscopic remission. While the efficacy of anti-TNFα agents achieving clinical remission has been established, evidence suggests that deep remission (DR) provides more durable remission [3–5]. Newer guidelines provided by the American College of Gastroenterology (ACG) and the International Organization for the Study of Inflammatory Bowel Disease (IOIBD) recommend mucosal healing with clinical remission as preferred treatment targets in UC and CD [32–34]. With the introduction of newer therapies such as ustekinumab, vedolizumab, tofacitinib, in addition to anti-TNFα agents, patients and gastroenterologists have more personalized treatment options suitable for long-term use. Therapeutics should be continued despite achieving deep remission, as withdrawal of therapy after achieving DR is associated with high rate of relapse [2]. Therefore, while efficacy of an agent is important, other factors including side-effect profile, cost, clinician experience, patient preference and comorbidities, and availability should be considered [35].

Anti-TNFα agents, the oldest and most well-studied biologic class in the treatment of IBD, carry multiple advantages over alternative biologics. In addition to their superior clinical efficacy, long-term outcomes and side-effect profiles are well described, and systemic effect enables the concurrent treatment of rheumatologic diseases. Furthermore, infliximab is available as biosimilars and adalimumab allows the option of administration via injectables [36, 37]. With regards to efficacy, a 2018 meta-analysis of RCTs estimated the efficacy of infliximab and adalimumab in achieving remission in CD [38]. Furthermore, a more recent 2020 review and network meta-analysis of RCTs estimated outcomes consistent with deep remission in UC using infliximab, adalimumab, and ustekinumab [7].

Real-world data (RWD), though acquired via cohort studies rather than randomized controlled trials, offers complementary information, providing generalizable clinical efficacy that can be compared to results reported by RCTs. Although considered to provide lower quality evidence, utility of RWD has recently been demonstrated by the VICTORY consortium, established to evaluate the efficacy of vedolizumab in CD and UC patients based on RWD gathered retrospectively from multiple institutions [39, 40]. GEMINI 1 reported a 41.8% to 44.8% rate of remission (Mayo <=2, no subscore >1) at 52 weeks, similar to the 41% rate of endoscopic remission (Mayo subscore = 0 ), clinical remission rate of 51%, and deep remission rate of 30% at 1 year follow-up reported by the VICTORY Consortium [40, 41]. While significant differences in study design and patient enrollment exist between GEMINI and VICTORY precluding direct comparison, the findings highlight the relevance of RWD for clinical decision-making and for directing future therapeutic research.

RWD has even been incorporated into recent guidelines published by the British Society of Gastroenterology and the United Arab Emirates consensus paper on diagnoses and management of IBD. These guidelines describe similar rates of clinical remission in UC treated with golimumab in both RWD sources and RCTs. There were further examples of similar outcomes derived from both data sources with regards to the efficacy of vedolizumab in UC, and separately the efficacy of adalimumab in UC [42, 43].

Our meta-analysis of fifteen real world studies of anti-TNFα use in CD and UC demonstrates that RWD DR rates supplement rates reported in existing phase III trial data and provides data in the setting of a potential efficacy-effectiveness gap. Though no clinically significant difference can be derived from the data, we observed a modestly higher observed rate of DR in UC in real-world studies. We report a DR rate of 48.6% at 1 year in CD using RWD, providing similar results compared to a previous meta-analysis of RCTs [38]. These results corroborate the findings of prior RCTs with regards to efficacy of anti-TNFs. An additional observation was that the rate of DR after 1 year of treatment was higher than earlier time points; following this peak, DR rates diminished by 2 years, suggesting that the greatest therapeutic benefit from anti-TNFα may be realized within the first 12 months. In sub-analysis, the rate of DR in anti-TNFα naïve patients at 1 year was 47.2% (95% CI 34.5–60.4%), similar to the DR rate at 1 year in all patients. Finally, we observe a small increase in the rate of deep remission when only including studies that evaluated response to infliximab.

This meta-analysis with systematic review is the first to comprehensively report DR with anti-TNFα agents based on RWD, using a strictly pre-defined definition of DR as clinical remission combined with endoscopic remission. We thoroughly reviewed the literature by incorporating results from Pubmed and EMBASE in addition to conference abstracts and review of references from publications. We additionally report remission rates at predefined time points. The inclusion of only RWD provides clinical effectiveness data in clinical practice settings, complementary and comparable to results reported by RCTs [11]. We anticipate the findings will help guide clinical decision making and elucidate the generalizability of these treatments to diverse and heterogenous populations.

There are several limitations. Constrained by available studies, we could not directly compare differences in DR rates between CD and UC. The limitation in number of available studies also precluded analysis of CD and UC at the 8 week, 6 month, and 2 year time points. Most studies utilized infliximab, therefore we were unable to provide a head-to-head comparison of biologic agents. We attempted to account for heterogeneity of biologics with additional analyses including only studies conducted with infliximab. Furthermore, paucity of available publications precluded the inclusion of newer therapeutic options. Adverse events were poorly reported in the included studies and were not able to be addressed in this analysis. We limited our search to English language publications, potentially introducing language bias into our results. Additionally, we recognize that there are varying definitions and sources of RWD, and therefore elected to use definitions and sources similar to those used in recent meta-analyses of RWD [39, 40]. Heterogeneity attributing to study design, use of cohort studies rather than RCTs, varying severity of disease in included patients, variations in concomitant medication usage, and differences in defining DR and endoscopic remission were expected given the utilization of RWD. The retrospective nature of some included studies also poses risk of bias, in particular with retrospective calculation of CDAI in patients with CD. Given the non-randomized nature of the included studies, there is significant risk of selection bias and potential confounders within individual studies. Our assessment is that the certainty of our findings are consistent with a low GRADE certainty rating given uncertainty of how biases may have influenced our results. This is due to the observational nature of the studies included which generated real-world data.

## Conclusions

We share the findings of a systematic review with meta-analysis of real-world data which evaluates deep remission from anti-TNFα utilization. The literature reviewed provides evidence that among IBD patients the application of an anti-TNF leads to the highest rate of deep remission at 52-weeks with an estimated 45% reaching this target. This finding reflects real-world data collected from literature review from 2012 to July 2019. Future systematic reviews focusing on the outcome of deep remission with the use of other novel targeted therapeutics are needed and can validate their efficacy in day-to-day practice.

## Supplementary Information

**Additional file 1. Table S1.** PRISMA statement manuscript checklist. **Table S2**. PRISMA statement abstract checklist. **Table S3**. Study heterogeneity. **Table S4**. Newcastle Ottawa Scale risk of bias scores.

**Additional file 2. Appendix 1.** Meta-analysis search strategy.

**Additional file 3: Fig. S1**. Sensitivity analysis removing studies with patients in clinical remission.

**Additional file 4: Fig. S2**. Funnel plot of studies included in meta-analysis for publication bias assessment.

## Data Availability

All data generated or analysed during this study are included in this published article and its supplementary information files.

## Abbreviations

CR: Clinical remission
DR: Deep remission
ER: Endoscopic remission
MH: Mucosal healing
RCT: Randomized control trial
RWD: Real-world data
