# Supplementary material for: Systematic review and meta-analysis: real-world data rates of deep remission with anti-TNFα in inflammatory bowel disease
Source: BMC Gastroenterol. 2021 Aug 3;21:312. doi: 10.1186/s12876-021-01883-6 (PMC8335971; doi:10.1186/s12876-021-01883-6)
Supplement: Supplementary file 2 — Additional file 2. Appendix 1. Meta-analysis search strategy. [file 12876_2021_1883_MOESM2_ESM.docx]

Appendix 1. Search Strategy

**Pubmed/MEDLINE**

The following search strategy implemented on July 8, 2019 retrieved 2,605 references:

("inflammatory bowel disease"[All Fields] OR "IBD"[All Fields] OR "crohn*"[All Fields] OR "ulcerative colitis"[All Fields] OR "CD"[All Fields] OR "UC"[All Fields] OR "colitis"[All Fields]) AND ("mucosal healing"[All Fields] OR "deep remission"[All Fields] OR "complete remission"[All Fields] OR "full remission"[All Fields] OR "endoscopic remission"[All Fields])

**EMBASE**

The following search strategy implemented on July 8, 2019 retrieved 5,882 references:

('inflammatory bowel disease'/exp OR 'inflammatory bowel disease' OR 'ibd' OR 'crohn*' OR 'ulcerative colitis'/exp OR 'ulcerative colitis' OR 'cd'/exp OR 'cd' OR 'uc' OR 'colitis'/exp OR 'colitis') AND ('mucosal healing'/exp OR 'mucosal healing' OR 'deep remission' OR 'complete remission'/exp OR 'complete remission' OR 'full remission' OR 'endoscopic remission')

An updated search using the strategy above from 7/8/19 to 4/25/21 yielded 1722 publications (596 Pubmed/MEDLINE, 1126 EMBASE). 93 publications underwent text review; none included data meeting inclusion criteria. This search and review was carried out by one author (BZ).
