# Supplementary figures and images for: Systematic review and meta-analysis: real-world data rates of deep remission with anti-TNFα in inflammatory bowel disease
Source: BMC Gastroenterol. 2021 Aug 3;21:312. doi: 10.1186/s12876-021-01883-6 (PMC8335971; doi:10.1186/s12876-021-01883-6)

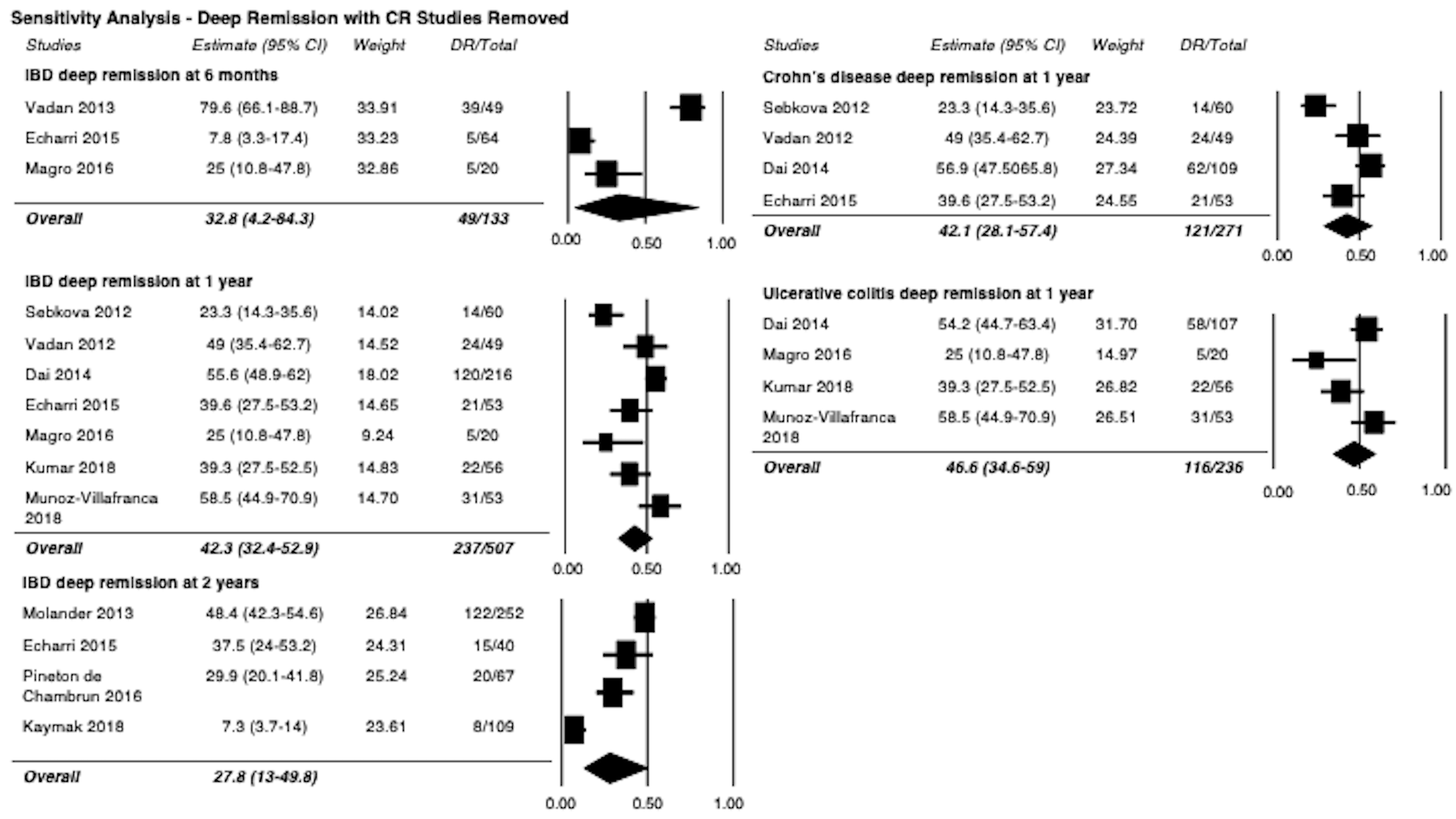

Supplement: Supplementary file 3 — Additional file 3: Fig. S1. Sensitivity analysis removing studies with patients in clinical remission. [file 12876_2021_1883_MOESM3_ESM.tiff]

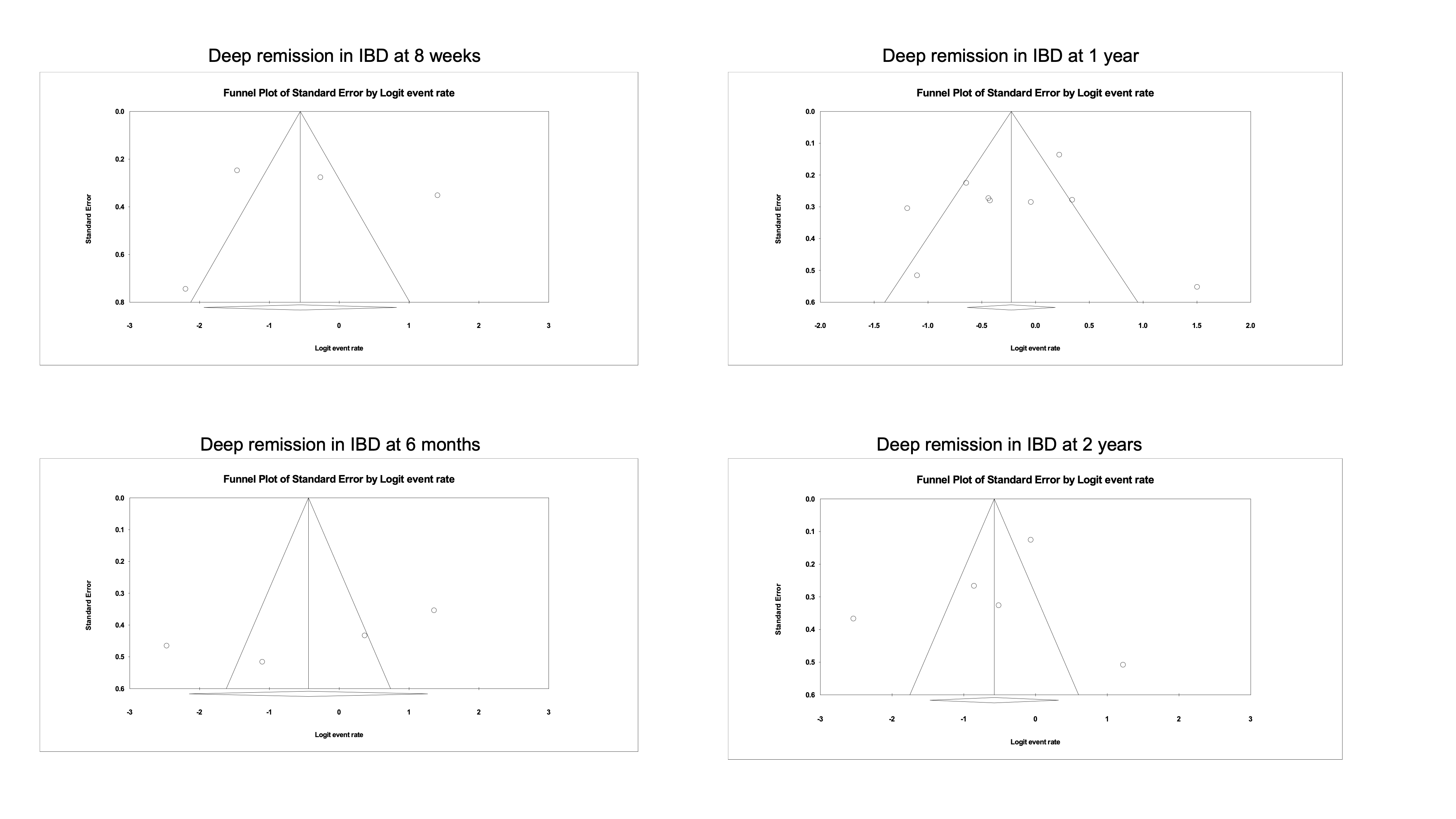

Supplement: Supplementary file 4 — Additional file 4: Fig. S2. Funnel plot of studies included in meta-analysis for publication bias assessment. [file 12876_2021_1883_MOESM4_ESM.tiff]
